# Diagnostic process of cancer in a health system without gatekeeping: a single centre survey analysis

**DOI:** 10.1017/S146342362400029X

**Published:** 2024-10-17

**Authors:** Ayşenur Duman Dilbaz, Saliha Serap Çifçili

**Affiliations:** 1 Medical Faculty, Department of Medical Education, Karadeniz Technical University, Trabzon, Turkey; 2 Medical Faculty, Department of Family Medicine, Marmara University, Istanbul, Turkey; 3 Medical Faculty, Eastern Mediterranean University, Famagusta, Northern Cyprus

**Keywords:** cancer diagnosis, diagnostic interval, patient delay, primary care, referral system

## Abstract

**Introduction:**

It is essential to increase the rates of early diagnosis in cancer control, and the diagnostic process needs to be improved to achieve this goal. Previous studies showed that in countries where there is a gatekeeping system, there might be a delay in cancer diagnosis. Our aim is to examine the process of cancer diagnosis in a healthcare system without gatekeeping.

**Method:**

A quantitative descriptive study has been conducted in various outpatient clinics of Pendik Training and Research Hospital, between 1 February and 31 May 2019, with individuals aged over 18 and diagnosed with cancer in the last six months. The data was collected through a questionnaire filled in by face-to-face interview method. Patient’s socio-economic characteristics, their symptoms at the time of the diagnosis and the diagnosis process were questioned.

**Result:**

The median diagnostic interval was 30 days (min–max 1–365), and the median patient interval was 60 (1–600) days. Patients pointed out that the diagnostic tests, especially the pathology reporting process, caused the diagnostic interval to be prolonged. Of the patients, 84% (*n* 135) stated that they did not consider their symptoms as a sign of serious illness. The patient interval was shortest with symptoms of haematuria and haematochezia and longest with dysuria and change in bladder habit.

**Discussion:**

The study examined the diagnosis process in our health system, where patients can apply for health services at any stage. The results showed that there were no superior outcomes to those observed in primary care-led health systems. Patients reported that waiting times for medical tests led to prolongation of the diagnosis time. Cancer awareness of patients should also be increased to shorten patient admission times.

## Introduction

Cancer is a significant public health problem due to its increasing incidence and high mortality rate. Cancer control programmes are being developed around the world, focusing on increasing screening rates to improve patient outcomes and reduce the financial burden (Ferlay *et al.*, 2021). One of the essential features of primary care is to serve as the initial point of contact for patients within the healthcare system. Due to its comprehensive, continuous and community-based nature, primary care is well-positioned to play a pivotal role in the early diagnosis of cancer. Primary care also facilitates early diagnosis by acting as a central point for cancer risk assessments and cancer screening (Hamilton, 2010; Emery *et al.*, 2013).

In countries where gatekeeping is implemented, patients can access advanced diagnostic methods and specialists if they are referred by primary care workers. In this type of system, the function of family physicians is called as ‘gatekeeper’. However, concerns have been raised in Europe, where gatekeeping is a common practice, that primary care referral times may result in delays in cancer diagnosis (Garrido *et al*., 2011; Lyratzopoulos *et al*., 2014; Singh *et al.*, 2017). Consequently, methods have been developed, such as the ‘fast-track system’ and the ‘two-week rule’, which facilitate accelerated diagnosis times by enabling patients to access upper-level care more promptly. Improvements have been reported in timely cancer diagnosis as a result of these measures (Meechan *et al.*, 2012; Holtedahl, 2020). This raises the question of whether the diagnosis time is shorter in health systems, where there is no gatekeeping or referral system, allowing patients to directly admit to secondary or tertiary health care. In Turkey, there is no referral system; therefore, the healthcare system is poorly coordinated, and patients can be admitted to any healthcare institution and specialty directly (Akman *et al.*, 2017). According to our literature search, there is no convincing evidence to accept or reject the role of the gatekeeping effect in cancer diagnosis.

Previous studies showed that age, education and income levels of patients affect the diagnostic process and are related to the cancer stage at the time of diagnosis (Macleod *et al.*, 2009). In addition, the types of symptoms, the way patients make sense of their symptoms and their beliefs affect the behaviour of seeking medical help (Smith *et al*., 2005; Molassiotis *et al.*, 2010). In order to increase the rate of early diagnosis, the diagnostic process needs to be well understood, and the causes of prolonged diagnosis should be identified. In our country, there is very limited data on the cancer diagnostic process.

In this study, we aimed to determine whether the duration of time to cancer diagnosis is reduced in a healthcare system without gatekeeping.

## Method

A quantitative descriptive study was carried out. Data was collected from various outpatient polyclinics of Marmara University Medical Faculty Pendik Research and Training Hospital (Istanbul) between 1 February and 31 May 2019.

As it is rare to encounter patients with low-prevalence diseases such as cancer in primary care, we conducted our study in a tertiary health centre to reach a larger patient population. In interviews, patients were asked whether they had any contact with primary care during the process of cancer diagnosis, from the onset of symptoms to the final diagnosis. We believe that our approach will not result in any bias due to the permeability between levels of the health system, given the lack of gatekeeping in our country.

The study included voluntary participants who were 18 years of age or older and had been diagnosed with cancer in the previous six months. Since the convenience sampling method was used, we didn’t calculate any sample size. Our country has a cancer registry system that is protected by the Ministry of Health. As it contains confidential patient information, we thought it would be unethical to use this data without obtaining individual patient consent. Given these ethical concerns, we preferred to collect data by interviewing the patients.

The patients were interviewed in various outpatient clinics over a period of three months, four days a week. We visited the medical oncology, gynaecologic oncology and urologic oncology outpatient clinics on the first three days each week. On the remaining day, we alternately visited the otolaryngology, pulmonology, thoracic surgery, general surgery and family medicine outpatient clinics. Patients who completed their doctor’s interview in the outpatient clinic were invited to participate in the study. Face-to-face interviews were conducted in a suitable room with patients who gave their informed consent.

A questionnaire form was prepared for data collection. This form was filled out by the researchers during the interviews. Researchers asked patients questions and noted responses. There was no need for a pilot study as the questions were asked face to face and explanations were given if they were not understood.

The World Health Organization (WHO) outlines the process of cancer diagnosis in three intervals and defines the period from the onset of symptoms to the patient’s first contact with a healthcare facility as the ‘patient interval’. The time from this initial contact to referral to secondary or tertiary care is termed the ‘primary care interval’. Finally, the duration from referral to the definitive diagnosis is referred to as the ‘diagnostic interval’ or ‘secondary care interval’ (World Health Organization, 2017). Based on this outline, we examined the patient interval and the diagnostic interval in our study. However, as there is no referral system in our country, we examined the diagnostic interval as ‘the time from the first suspicion of cancer in the patient by any level of health care to the definitive diagnosis’.

The questionnaire covered patients’ demographic and socio-economic characteristics, diagnosis and stage at diagnosis, preferred healthcare level and symptoms at diagnosis. It was particularly important to know whether the patients had an ‘alarm symptom’ at the time of diagnosis. Symptoms that are suspicious and have a predictive value for careful examination in the diagnosis of cancer are called ‘alarm symptoms’ and can shorten the diagnostic process (Hippisley-Cox and Coupland, 2013a, 2013b). They were therefore specifically asked about and recorded. To better understand the patient interval, the questionnaire also included the following items: how they made sense of their symptoms, actions they took after noticing symptoms and the healthcare level they went to with symptoms and the healthcare level where cancer was first suspected. The factors prolonging the interval according to the patients’ perception were also questioned.

The SPSS 11.5 (Statistical Package for the Social Sciences) programme was used for data analysis. In the analyses, *P*-values below 0.05 were considered significant. In the analysis of descriptive data, frequency values, mean values for continuous variables and median values were calculated when the data wasn’t normally distributed. Answers to open-ended questions were grouped and analysed. In comparative analyses, the chi-square test or Kruskal–Wallis H test was used for categorical variables. Student *t*-test was used for comparative analysis of two continuous variables. One-way analysis of variance (ANOVA) was used when there were more than two continuous variables. When parametric assumptions were not met, the Mann–Whitney U test was used, comparing two variables and Kruskal–Wallis when there were more than two variables. In correlation analysis of continuous variables, the Pearson correlation test is used for normally distributed data, while the Spearman correlation test is preferred for non-normally distributed data.

Ethics committee approval was received from the Scientific Research Ethics Committee of Marmara University Faculty of Medicine **(protocol number: 09.2019.183, date: 01.02.2019)**.

## Results

The number of patients who agreed to participate in our study was 176. The mean age of the participants was 55 (SD = 13, minimum = 18, maximum = 88). Other socio-demographic characteristics of the participants are shown in Table 1.

**Table 1.** Socio-demographic characteristics of the participants

Characteristics		Frequency
Sex	Female	%40 (*n* = 70)
	Male	%60 (*n* = 106)
Marital status	Married	%78 (*n* = 138)
	Single	%5 (*n* = 9)
	Divorced/widowed	%17 (*n* = 39)
Education	Illiterate	%8 (*n* = 14)
	Literate	%2 (*n* = 49)
	Primary/junior high school	%60 (*n* = 105)
	High school	%16 (*n* = 29)
	University	%14 (*n* = 21)
Income level	Low	%24 (*n* = 43)
	Medium	%65 (*n* = 112)
	High	%12 (*n* = 21)

In our study, 21% (*n* = 37) of the patients were diagnosed in the early stage, 51.1% (*n* = 90) in the intermediate stage and 27.8% (*n* = 49) in the advanced stage. The mean age of patients diagnosed in the advanced stage of the disease was 64.4 years (SD = 11), while those diagnosed in the early/intermediate stage were, on average, 58.2 (SD = 13) years old. There was a statistically significant difference between the two groups (independent samples *t*-test, *P* = 0.006). No significant correlation was found between other socio-demographic characteristics of the patients and their stage at the time of cancer diagnosis. The diagnostic stage does not differ significantly between the cancer types (*P* = 0.14).

The median value of the diagnostic interval for all patients was found to be 30 days (mean = 39.7, SD = 40, min–max = 1–365) days. The diagnostic interval in 56 patients (33%) was above 30 days. There was no correlation between the diagnostic interval and the stage at the time of diagnosis (Spearman correlation test, *P* = 0.59, rho = 0,048).

The most common 10 cancer types and the diagnostic intervals are given in Table 2. No significant difference was found between cancer types in terms of diagnostic interval (Kruskal–Wallis H test, *P* = 0.29).

**Table 2.** The 10 most common cancers and diagnostic intervals in our study

Types of cancer	%	Diagnostic interval median value (days)
1. Lung	%19.3 (*n* = 34)	30 (min–max 1–20)
2. Colorectal	%18.2 (*n* = 32)	30 (min–max 2–150)
3. Breast	%12.5 (*n* = 22)	30 (min–max 7–120)
4. Stomach	%12.5 (*n* = 22)	30 (min–max 3–90)
5. Pancreas	%6.3 (*n* = 11)	21 (min–max 5–60)
6. Prostate	%5.7 (*n* = 10)	90 (min–max 7–120)
7. Endometrium	%4.5 (*n* = 8)	30 (min–max 7–120)
8. Over	%4 (*n* = 7)	25 (min–max 15–30)
9. Cervix	%2.8 (*n* = 5)	21 (min–max 10–365)
10. Bladder	%2.3 (*n* = 4)	18 (min–max 5–90)

There were 160 patients with at least one symptom at the time of diagnosis (91%). Table 3 shows the behaviours of patients after noticing their symptoms. The symptom types of the patients and the patient intervals are given in Table 4. The median value of the patient interval was 60 days (mean = 90, min–max = 1–600 days). Although the number of symptoms present at the time of diagnosis is at most four for a patient, there were 16 (9.1%) patients with no symptoms as well. There was no significant relationship between the patient interval and gender (Student *t*-test, *P* = 0.218), income and education levels (one-way ANOVA test, *P* = 0.6 and *P* = 0.149). Of the 16 patients with no symptoms at the time of diagnosis, 6 stated that their diagnostic process started with screening and 10 with abnormalities detected in examinations performed for other reasons.

**Table 3.** Behaviours of patients after noticing their symptoms

Responses	N	%
I did not think that my complaints could be a sign of a severe illness.	135	%84.3
I waited for a while, thinking that my complaints would go away on their own.	72	%45.0
I did not see my complaints as a symptom of illness. I thought that they were due to natural causes.	33	%20.6
I tried to treat my complaints by myself or by consulting my environment (using drugs, herbal products, applying alternative medicine methods, etc.).	30	%19
I thought that my complaints were related to my existing chronic diseases.	27	%17

**Table 4.** Symptoms present at the time of diagnosis and time from symptom onset to first admission (patient intervals)

Symptoms	N	Patient interval/median days (min–max)	Symptoms	N	Patient interval/median days (min–max)
Abdominal pain	33	60 (1–180)	Night sweats*	6	30 (3–120)
Weight loss*	31	60 (7–365)	Jaundice/yellowing	6	30 (5–365)
Palpable mass*	27	30 (1–365)	Haematuria*	5	10 (3–30)
Change in bowel habit*	17	60 (7–600)	Low back pain	5	60 (4–150)
Fatigue	16	60 (20–365)	Loss of appetite*	5	30 (20–90)
Nausea/vomiting	16	12 (1–365)	Haemoptysis*	5	30 (3–270)
Cough*	15	15 (4–365)	Pain/cramps in muscles	4	197 (15–365)
Back pain	13	60 (2–365)	Difficulty swallowing*	4	75 (30–365)
Abnormal vaginal bleeding*	12	30 (1–365)	Change in bladder habit*	3	120 (60–365)
Haematochezia*	11	2 (1–240)	Dizziness	3	60 (7–180)
Abdominal bloating	9	60 (5–180)	Itching	3	30 (10–180)
Shortness of breath	7	30 (1–365)	Chest pain	3	15 (10–180)
Change in the skin lesions/moles*	6	25 (2–180)	Headache	2	93 (7–180)
Dysuria	6	120 (7–240)	Lower extremity oedema	2	75 (60–90)

* Alarm symptoms Hippisley-Cox and Coupland (2013a; 2013b).

Alarm symptoms present at the time of diagnosis, and their frequencies and the symptom-patient intervals are given in Table 4. Of the patients who had at least one symptom, 71% (*n* = 114) had at least one alarm symptom.

The patients were asked about the processes that they thought had a negative effect on the diagnostic interval, and the most frequent answer was the pathology reporting time in 27% (*n* = 47) of patients. Other responses and their duration are given in Table 5.

**Table 5.** Procedures that patients believe prolong the diagnostic interval

Processes	N	Mean/day
Pathology reporting time	47 (%27)	29 ± 12 (median 30)
Tissue sampling appointment waiting time	15 (%9)	28 ± 21 (median 21)
Outpatient clinic appointment waiting time	11 (%6)	18 ± 10 (median 20)
MRI reporting time	11 (%6)	17 ± 8 (median 15)
PET-CT reporting time	11 (%6)	20 ± 10 (median 20)
Time taken to make an outpatient clinic appointment.	5 (%3)	34.4 ± 32 (median 30)
USG appointment waiting time	5 (%3)	30 ± 18 (median 30)

MRI: Magnetic Resonance Imaging, PET-CT: Poitron Electron Tomography-Computed Tomography, USG: Ultrasonography.

In addition, we have asked about the healthcare facility they prefer to admit when they have any kind of symptoms. Of the participants, 45% (*n* = 79) stated they would choose primary care, and 44% (*n* = 78) would choose to consult a specialist physician (secondary or tertiary care). However, only 19% of the patients (*n* = 34) initially admitted to primary care when they noticed the symptoms. The first step in which the patients were suspected of cancer was the primary care clinic in 9% (*n* = 18) patients, the secondary care in 45% (*n* = 79) and the tertiary care in 29% (*n* = 51) and the emergency department in 16% (*n* = 46) of the patients. No significant difference was found between the healthcare centre at which the patients were suspected of having cancer for the first time and the stage of cancer at the time of the diagnosis (chi-square test, *P* = 0.693) and with the diagnostic interval (Kruskal–Wallis test, *P* = 0.112).

## Discussion

In the present study, we examined the process of diagnosing cancer in a healthcare system in which primary care does not play the gatekeeper role. Our main findings are that the median diagnostic interval was 30 days (min–max = 1–365) and the patient interval was 60 days (min–max = 1–600). The results showed that most patients did not relate their symptoms to a serious illness; many waited for them to go away on their own or tried to treat them on their own. The symptom types of the patients also affect the patient interval. Our study is one of the rare studies in which patients’ opinions are taken in terms of the factors that cause the prolongation of the cancer diagnosis process. In their opinions, patients pointed diagnostic tests as a significant factor contributing to the prolonged diagnosis process, particularly pathology reporting times.

There is no common figure in the world regarding how many days the cancer diagnosis process will be considered as a delay. WHO recommends that the time from referral to definitive diagnosis shouldn’t exceed 30 days, with a target of at least 80% of patients should be diagnosed in this period (World Health Organization, 2017). In our study, we considered the diagnostic interval to start from the first suspicion of cancer, not primary care referral since there is no gatekeeping in our country. The diagnostic interval started at the secondary or tertiary care for 74% (*n* = 130) of the patients, but still 33% (*n* = 56) of the patients had a diagnostic interval longer than 30 days. Although patients can easily reach the specialist care, our study shows that the diagnosis interval is longer than WHO’s recommendation.

In Murchie *et al*.’s study, in three countries in Europe where primary care is coordinating the health system, the mean time from the first GP admission to the definitive diagnosis was found to be 22 days in the Netherlands, 32 days in Sweden and 53.5 days in Scotland (Murchie *et al.*, 2012). Scotland’s primary care referral time and diagnosis time were significantly longer. Murchie *et al*. commented that research on the differences in time between these countries with similar health systems, based on health records, and the differences in keeping these records may affect the results of the study. In a review of studies conducted in 57 low- and middle-income countries, Brand *et al*. found that the median value of the diagnosis time (the time from the patient’s first admission to the definitive diagnosis) was 0.9 months (min–max = 0.6–2.8) (Brand *et al.*, 2019). These studies show that similar diagnostic intervals can be observed in countries with different health systems in the world. It is also evident that different diagnostic processes can be observed in countries that are in a similar context and have the same health system functioning. The studies demonstrate that each healthcare system should have its own distinctive characteristics and that isolating the impact of gatekeeping on the cancer diagnosis process is challenging. Within each country, other crucial factors that influence the diagnostic process in their respective contexts should also be identified.

The objective of the gatekeeping system in health care is to enhance efficiency by preventing unnecessary specialist examinations, unnecessary repeated medical tests and arbitrary multiple admissions. A study comparing health systems in European countries found that in countries without a referral system, patients without a chronic disease consulted physicians at the same rate as patients with chronic diseases. In addition, the referral rate of patients without chronic diseases was found to be lower in countries where gatekeeping was in place (Reibling and Wendt, 2010). In our country, it is known that unnecessary examinations and tests are carried out as a result of patients admitting to specialist care without being differentiated, admitting to specialists that are not suitable for their complaints and making more admissions than needed (Yardim and Uner, 2018; Çıraklı, 2020). In the current system, the utilisation of primary care by patients is also low. According to the Ministry of Health statistics, primary care utilisation rates in our country between 2016 and 2020 varied between 32% and 42% (Republic of Türkiye, Ministry of Health, 2020). In our study, the rate of primary care utilisation was found to be similarly low. Consequently, we believe our findings are similar of the average for Turkey.

The low utilisation of primary care in our country results in a high workload at the secondary and tertiary care (General Directorate of Health Information Systems, Republic of Türkiye, Ministry of Health, 2020). Despite an increase in the number of physicians over recent years, the admission rate of patients has also risen. To respond to the increasing patient load, the outpatient clinic hours of physicians are being increased, and the time allocated for examination is getting shorter (Yardım *et al.*, 2017; Çıraklı, 2020). Increased workloads in hospitals also result in inadequate numbers of staff, technical infrastructure, medical equipment and supplies. It is therefore not surprising that in our study, when patients were asked about their experience of the diagnostic process, they emphasised that the diagnostic imaging and pathology reporting processes prolonged the diagnostic interval. According to the Organisation for Economic Co-operation and Development (OECD), the number of imaging devices per capita in our country is comparable to the European average. However, the rate of diagnostic tests per patient is higher than in Europe and is increasing (*OECD Data*, 2023). This may be a consequence of the reduced time available for history taking and examination, which may lead doctors to order more diagnostic tests in an effort not to miss a serious condition. Implementing a referral system and increasing the use of primary care in our country could help reduce the burden on hospitals. This would improve the process of cancer diagnosis and increase the rate of early diagnosis.

Another significant interval in the cancer diagnosis process is the patient interval. In our study, 91% of patients had at least one symptom, but the median time patients waited before being admitted to a health service was 60 days. Although there is no agreed figure in the world for patient interval, WHO states that the patient interval shouldn’t exceed 30 days (World Health Organization, 2017). Several studies showed that the duration of the patient interval may be longer in patients with older age, lower socio-economic status and educational level. Non-specific symptoms such as nausea and fatigue are also a cause of late presentation to health care. In addition, how patients make sense of their symptoms and their feelings, such as shame and anxiety, also influences the patient interval (Smith *et al*., 2005; Macleod *et al.*, 2009; Molassiotis *et al.*, 2010). In our study, no significant correlation was found between the demographic, socio-economic and educational level of the patients and the patient interval. We believe that this situation is related to the fact that access to health care in our country is easy and health insurance is comprehensive so that all segments of society can benefit from healthcare services (Yardim and Uner, 2018).

Among our patients who took part in the study, 84% said they did not associate their symptoms with a serious illness. Most patients waited for their symptoms to resolve on their own. Some of them tried to treat themselves or attributed the symptoms to the chronic conditions they had. These findings suggest that patients’ knowledge and awareness about the symptoms of cancer are low. In the systematic review examining cancer research in countries with low- and middle-income levels, Brand *et al*. explained that patients’ health literacy, symptom knowledge and the way they interpret symptoms are significant barriers to early diagnosis (Brand *et al.*, 2019). Pedersen *et al*. conducted a study in five developed countries and similarly stated that the patients’ poor recognition of cancer symptoms and their negative beliefs led to a prolonged diagnosis period (Pedersen *et al.*, 2018). As shown in these studies, we believe that longer patient intervals in our study are also associated with low patient awareness of cancer.

In several previous studies, painful or bleeding symptoms that affect daily life led to faster patient intervals, and patients with non-specific symptoms took longer to seek medical help (Nekhlyudov and Latosinsky, 2010; Whitaker *et al.*, 2014). Similarly, our participants’ patient intervals were shortest for haematochezia (two days) and longest for muscle cramps and pain (197 days). Symptoms that are bleeding, painful and affecting daily life seem to evoke a universal sense of importance. In our study, we found that many patients with alarm symptoms such as dysphagia waited a long time before presenting to healthcare facilities. Alarm symptoms are acknowledged to have significant predictive value in the diagnosis of cancer, and it is known that the rates of patient referral increase in the presence of alarm symptoms (Nekhlyudov and Latosinsky, 2010). Our participants’ low awareness of alarm symptoms may be the reason for their delay in seeking medical help. As cancer alarm symptoms can be recognised by healthcare professionals and trigger an acceleration of the diagnosis process, informing the public about alarm symptoms may improve the cancer diagnosis process.

## Limitations of the study

The research was conducted in a single centre using convenience sampling, which may introduce selection bias, so the results cannot be generalised to the whole population. One of the limitations of our study is that the participants comprised only outpatient clinics related to cancer. Because the data was collected through questionnaires with questions asked to the patients retrospectively, recall bias may have occurred. To limit this bias, a time limit was set by including only the patients diagnosed within the last six months.

## Conclusion

Our study shows that the cancer diagnosis process is no better in a healthcare system without gatekeeping. Without gatekeeping, the usage of primary care is very limited, which leads to excessive use of hospital care and possibly prolongs the diagnostic interval. It seems it might be helpful to increase awareness among patients about cancer symptoms. To clearly demonstrate the effect of gatekeeping in cancer diagnosis, future studies are needed that consider confounding factors affecting the process, such as patient delay and diagnostic testing, which also emerged in our study.

## References

[ref1] Akman M , Sakarya S , Sargın M, Ünlüoğlu İ , Eğici MT , Boerma WGW and Schäfer WLA (2017) Changes in primary care provision in Turkey: a comparison of 1993 and 2012. Health Policy 121, 197–206. Available at: 10.1016/J.HEALTHPOL.2016.11.016 27932252

[ref2] Brand NR , Qu LG, Chao A and Illbawi AM (2019) Delays and barriers to cancer care in low- and middle-income countries: a systematic review. The Oncologist 24, e1371–e1380. Available at: 10.1634/THEONCOLOGIST.2019-0057 31387949 PMC6975966

[ref3] Çıraklı Ü (2020) Comparison of average patient examination times per capita between 2002–2018 in 18 OECD countries. Journal of Healthcare Quality and Accreditation 3, 43–54. Retrieved 17 January 2023 from https://dergipark.org.tr/en/pub/jhqa/issue/55443/757279

[ref4] Emery JD, Shaw K, Williams B, Mazza D, Fallon-Ferguson J, Varlow M and Trevna LJ (2013) The role of primary care in early detection and follow-up of cancer. Nature Reviews Clinical Oncology 11, 38–48. Available at: 10.1038/nrclinonc.2013.212 24247164

[ref5] Ferlay J, Colombet M, Soerjomataram I, Parkin DM, Piñeros M, Znaor A and Bray F (2021) Cancer statistics for the year 2020: an overview. International Journal of Cancer 149, 778–789. Available at: 10.1002/IJC.33588 33818764

[ref6] Garrido MV , Zentner A and Busse R (2011) The effects of gatekeeping: a systematic review of the literature. Scandinavian Journal of Primary Health Care 29, 28–38. Available at: 10.3109/02813432.2010.537015 21192758 PMC3347935

[ref20] General Directorate of Health Information Systems, Republic of Türkiye, Ministry of Health (2020) Utilization of healthcare services: 148–190. In: Bora Başara B, Soytutan Çağlar I, Aygün A, Özdemir TA and Kulalı B (Eds). Health statistics yearbook. Ankara: Ministary of Health. Retrieved 17 December 2023 from https://www.saglik.gov.tr/Eklenti/43400/0/siy2020-eng-26052022pdf.pdf

[ref7] Hamilton W (2010) Cancer diagnosis in primary care. British Journal of General Practice, 60, 121–128. Available at: 10.3399/BJGP10X483175. PMC2814263 20132704

[ref9] Hippisley-Cox J and Coupland C (2013a) Symptoms and risk factors to identify men with suspected cancer in primary care: derivation and validation of an algorithm’, British Journal of General Practice 63, e1–e10. Available at: 10.3399/BJGP13X660724 PMC3529287 23336443

[ref10] Hippisley-Cox J and Coupland C (2013b) Symptoms and risk factors to identify women with suspected cancer in primary care: derivation and validation of an algorithm. British Journal of General Practice 63, e11–e21. Available at: 10.3399/BJGP13X660733 PMC3529288 23336450

[ref11] Holtedahl K (2020) Challenges in early diagnosis of cancer: the fast track. Scandinavian Journal of Primary Health Care 38, 251–252. Available at: 10.1080/02813432.2020.1794415 32791936 PMC7470137

[ref12] Lyratzopoulos G , Wardle J and Rubin G (2014) Rethinking diagnostic delay in cancer: how difficult is the diagnosis? BMJ 349, S92–S101. Available at: 10.1136/BMJ.G7400 25491791

[ref13] Macleod U, Mitchell ED, Macdonald S and Ramirez AJ (2009) Risk factors for delayed presentation and referral of symptomatic cancer: evidence for common cancers. British Journal of Cancer 101, S92–S101. Available at: 10.1038/sj.bjc.6605398 19956172 PMC2790698

[ref14] Meechan D, Gildea C, Hollingworth L, Richards MA, Riley D and Rubin G (2012) Variation in use of the 2-week referral pathway for suspected cancer: a cross-sectional analysis. British Journal of General Practice 62, e590–e597. Available at: 10.3399/BJGP12X654551 PMC3426597 22947579

[ref15] Molassiotis A, Wilson B, Brunton L and Chandler C (2010) Mapping patients’ experiences from initial change in health to cancer diagnosis: a qualitative exploration of patient and system factors mediating this process. European Journal of Cancer Care 19, 98–109. Available at: 10.1111/J.1365-2354.2008.01020.X 19552730

[ref16] Murchie P , Campbell NC, Delaney EK, Dinant G, Hannaford PC, Johansson L, Lee AJ, Rollano P and Spigt M (2012) Comparing diagnostic delay in cancer: a cross-sectional study in three European countries with primary care-led health care systems. Family Practice 29(1), 69–78. Available at: 10.1093/FAMPRA/CMR044 21828375

[ref17] Nekhlyudov L and Latosinsky S (2010) The interface of primary and oncology specialty care: from symptoms to diagnosis. JNCI Monographs 2010, 11–17. Available at: 10.1093/JNCIMONOGRAPHS/LGQ001 PMC3482946 20386049

[ref8] OECD Data, Indicator: Doctors (2023) Retrieved 17 January 2023 from https://data.oecd.org/healthres/doctors.htm

[ref18] Pedersen AF , Forbes L, Brain K, Hvidberg L, Wulff CN, Lagerlund M, Hajdarevic S, Quaife SL and Vedsted P (2018) Negative cancer beliefs, recognition of cancer symptoms and anticipated time to help-seeking: an international cancer benchmarking partnership (ICBP) study. BMC Cancer 18, 1–10. Available at: 10.1186/S12885-018-4287-8. 29609534 PMC5879768

[ref19] Reibling N and Wendt C (2010) Access regulation and utilization of healthcare services, *113*, 36. Retrieved 17 January 2023 from https://www.ssoar.info/ssoar/handle/document/19549

[ref21] Singh H , Schiff GD, Graber ML, Onakpoya I and Thompson MJ (2017) The global burden of diagnostic errors in primary care. BMJ Quality & Safety 26, 484–494. Available at: 10.1136/BMJQS-2016-005401 PMC5502242 27530239

[ref22] Smith LK , Pope C and Botha JL (2005) Patients’ help-seeking experiences and delay in cancer presentation: a qualitative synthesis. The Lancet 366, 825–831. Available at: 10.1016/S0140-6736(05)67030-4 16139657

[ref23] Whitaker KL , Scott SE, Winstanley K, Macleod U and Wardle J (2014) Attributions of cancer “Alarm” symptoms in a community sample. PLOS ONE 9, e114028. Available at: 10.1371/JOURNAL.PONE.0114028 25461959 PMC4252079

[ref24] World Health Organization (2017) Guide to Early Cancer Diagnosis. Geneva. Retrieved 17 December 2023 from https://www.afro.who.int/sites/default/files/2017-05/9789241511940-eng.pdf

[ref25] Yardım M and Eser E (2017) How many minutes should be reserved per patient in ambulatory care visits? Turkish Journal of Public Health 15, 58–67. Available at: 10.20518/TJPH.326827

[ref26] Yardim MS and Uner S (2018) Equity in access to care in the era of health system reforms in Turkey. Health Policy 122, 645–651. Available at: 10.1016/J.HEALTHPOL.2018.03.016 29598885

